# Lysine Acylation Modification Landscape of *Brucella abortus* Proteome and its Virulent Proteins

**DOI:** 10.3389/fcell.2022.839822

**Published:** 2022-03-01

**Authors:** Xi Zhang, Jingjing Chen, Qiao Dong, Jinying Zhu, Ruihao Peng, Chuanyu He, Yuzhuo Li, Ruiqi Lin, Pengfei Jiang, Min Zheng, Huan Zhang, Shiwei Liu, Zeliang Chen

**Affiliations:** ^1^ Key Laboratory of Livestock Infectious Diseases, Ministry of Education, Shenyang Agricultural University, Shenyang, China; ^2^ Department of Epidemiology, School of Public Health, Sun Yat-sen University, Guangzhou, China; ^3^ Department of Nephrology and Endocrinology, Wangjing Hospital, Chinese Academy of Chinese Medical Science, Beijing, China; ^4^ Innovative Institute of Zoonoses, Medical College, Inner Mongolia Minzu University, Tongliao, China

**Keywords:** *Brucella*, 2-hydroxyisobutyrylation, succinylation, crotonylation, acetylation, malonylation

## Abstract

The myriad of posttranslational modifications (PTMs) of proteins that occur in all living cells are crucial to all kinds of biological processes. *Brucella* is an intracellular parasitic bacterium that can cause chronic diseases in both humans and livestock. To reveal the relationship between PTMs and the virulence and survival of *Brucella*, we described the first comprehensive multiple PTM-omics atlas of *B. abortus* 2308. Five PTMs involving lysine, namely 2-hydroxyisobutyrylation, succinylation, crotonylation, acetylation, and malonylation were identified. Nearly 2,000 modified proteins were observed, and these proteins took part in many biological processes, with a variety of molecular functions. In addition, we detected many significant virulence factors of *Brucella* among the modified proteins. 10 of the 15 T4SS effector proteins were detected with one or more PTMs. Moreover, abundant PTMs were detected in other typical virulence factors. Considering the role of PTMs in various biological processes of *Brucella* virulence and survival, we propose that the virulence of *Brucella* is associated with the PTMs of proteins. Taken together, this study provides the first global survey of PTMs in *Brucella*. This is a prospective starting point for further functional analysis of PTMs during the survival of *Brucella* in hosts, interpretation of the function of *Brucella* proteins, and elucidation of the pathogenic mechanism of *Brucella*.

## Introduction

Although Brucellosis is known to seriously endanger the livestock industry, it is one of the most neglected prevalent zoonotic diseases. This disease is caused by *Brucella* species comprising intracellular Gram-negative bacteria that can survive and develop in different host cells. Colonization of macrophages and dendritic cells is particularly common, since *Brucella* can establish a replicative niche asymptomatically and escape the destruction of host cells (Archambaud et al., 2010), (Celli, 2019). Brucellosis is characterized by widespread aerosol transmission, and humans infected with brucellosis are mainly engaged in occupations where they deliver infected livestock or come into direct contact with unpasteurized animal products (Arroyo Carrera et al., 2006), (McDermott et al., 2013). *Brucella* infection generally causes flu-like symptoms, of which, fever is the most typical indication, and these clinical characteristics of brucellosis are commonly confused with those of other diseases. More than 500,000 cases of brucellosis are reported in humans annually (Seleem et al., 2008), especially in developing regions, such as Africa, the Middle East, South America, and Asia, where domestic screening and vaccination programs for livestock fail to control and eradicate the disease (Franc et al., 2018). The yearly numbers of brucellosis cases in China in 2018, 2019, and 2020 were 21,735; 44,036; and 47,245 respectively. Moreover, in November 2019, four students of the Lanzhou Veterinary Research Institute of the Chinese Academy of Agricultural Sciences were serologically positive for brucellosis. This incident was caused by the improper disposal of the A19 vaccine. As of 5 November 2020, a total of 6620 *Brucella* antibody-positive cases had been reported. Furthermore, due to the airborne transmission characteristics of brucellosis, *Brucella* is considered a biological weapon (Pappas et al., 2006), (Minor, 2015).

Virulent *Brucella* species cause systemic and chronic infections and have protein coats that protect their survival in cells. The pathogenic mechanism of *Brucella* does not depend on classical virulence factors, such as exotoxins, flagella, and capsules; in contrast, it depends largely on the ability of the bacteria to trigger their virulence mechanism, and select their physiological adaptation based on their interaction with the host (Skvortsova et al., 2018). Once the bacterium invades host cells, it forms a *Brucella* containing vacuole (BCV), enabling the bacteria to escape the host immune system and continue to survive by depending on the Type IV secretory system (T4SS) (Chandran, 2013). The T4SS is encoded by the VirB operon, whose syringe-like structure can secrete effector proteins (a total of 15 effector proteins have been identified to date) to help *Brucella* escape destruction by host cells (Caswell et al., 2012), (Ke et al., 2015), (Comerci et al., 2001). Among them, RicA interacts preferentially with host protein Rab2 and prevents the recruitment of Rab2 to the BCV, thereby affecting normal intracellular trafficking (de Barsy et al., 2011). BtpA and BtpB interfere with the TLR pathway to inhibit innate immune responses (Comerci et al., 2001). *Brucella* infection also impairs the process of host protein secretion that requires BspA, BspB, and BspF. Single or combined deletions of BspA, BspB, and BspF affect the ability of *Brucella* to replicate in macrophages and persist in the livers of infected mice (Myeni et al., 2013). Taken together, *Brucella* regulates secretory trafficking via multiple T4SS effector proteins which may synergistically promote the pathogenesis of *Brucella*. Multiple proteins participate in various pathways to co-regulate intracellular survival. Although much has been done to understand how the VirB system affects intracellular trafficking, the virulence process, and how this complicated secretion system is transcriptionally regulated, very poorly is known. The identity of the secreted/translocated proteins as well as the molecular mechanisms that modulate intracellular traffickin are also not well understood. Therefore, it is of great significance to study the function of proteins to clarify the pathogenic mechanism of *Brucella*.

Post-translational modification (PTM) of proteins, a typical epigenetic regulation, is considered to be the second vector that can transmit genetic information (Skvortsova et al., 2018). PTM of proteins contributes greatly to biodiversity and individual complexity and is closely involved in cellular regulation and disease occurrence. Therefore, the study of PTMs is crucial for pathogen research (Yang et al., 2014). Previous studies have reported several types of PTM, including lysine 2-hydroxyisobutyrylation (Dai et al., 2014), lysine succinylation (Zhang et al., 2011), lysine crotonylation (Tan et al., 2011), lysine acetylation (Cohen and Yao, 2004), lysine malonylation (Peng et al., 2011), lysine phosphorylation (Deutscher and Saier Jr, 2005), lysine butyrylation (Chen et al., 2007), and lysine lactylation (Zhang et al., 2019a). These modifications are responsible for different physiological functions. For example, lysine 2-hydroxyisobutyrylation occurs extensively in histones and plays an important regulatory role in the differentiation of sperm cells. Levels of lysine succinylation may cause morphological differences among Toxoplasma gondii, and lysine 2-hydroxyisobutyrylation affects the athletic ability of the parasite (Dai et al., 2014), (Huang et al., 2017). Lysine succinylation is common among species and is evolutionarily conserved. It can be dynamically adjusted with changes in the physiological environment, suggesting that this form of modification may have important functions in cells. For example, it reportedly plays a significant role in metabolic regulation, particularly in the tricarboxylic acid (TCA) cycle (Galván-Peña et al., 2019), (Sreedhar et al., 2020), (Zhou et al., 2019). Aflatoxins is the important virulence source of *Aspergillus flavus* (Hedayati et al., 2007). In one study, researchers mutated the succinylation site of the succinylase, NorA/Aad/Adh-2/NOR reductase/dehydrogenase (aflE), which is directly involved in aflatoxin biosynthesis, virulence and infectivity of the mutant were significantly reduced, suggesting that succinylation at the site affected aflatoxin synthesis by regulating the function of AflE, thus affecting the virulence and infectivity of *Aspergillus flavus* (Ren et al., 2018). Lysine crotonylation affects active transcription of promoter and enhancer regions, which are involved in regulating active genes of the neutral chromosomes in sperm cells during the anaphase of meiosis (Wan et al., 2019). Lysine acetylation is a evolutionarily conserved and highly abundant modification, and more than 20% of proteins in the mitochondria are known to be acetylated, which means that lysine acetylation may extensively affect various cellular physiological and biochemical functions (Kim et al., 2006). Lysine acetylation also can have a strong impact on the biochemical functions of proteins because the transfer of the acetyl group to lysine masks the positive charge, which is important for enzyme catalysis, protein-protein interactions and protein-DNA interactions (Glozak et al., 2005). In a previous study about *Mycobacterium tuberculosis* H37Ra strain, the results showed that acetylation of bacterial proteins affected the utilization of carbon source by bacteria, resulting in a slower growth rate (Liu et al., 2014). In *Enterococcus*, however, the results were reversed, suggesting that precise regulatory mechanisms may vary from species to species (Wang et al., 2010). In addition, studies on *Mycobacterium tuberculosis* and *Escherichia coli* have shown that acetylation of stress proteins can improve the ability of bacteria to resist heat stress (Liu et al., 2014) (Ma and Wood, 2011). Moreover, lysine acetylation can also affect colony morphology by regulating the activity of enzymes related to fatty acid metabolism, for example, H37RaΔMR_1161, is more granular than WT H37Ra (Liu et al., 2014). Lysine malonylation is associated with inflammatory signals in macrophages (Galván-Peña et al., 2019). Although most of the current PTM research has focused on histones, non-histone PTMs are also actively being explored. Previous studies have shown that the frequency of non-histone modifications is very high, and represent the major proportion of modified proteins in the cell (Cheng et al., 2009).

*Brucella* cause serious damage to animal husbandry industries and human health, but the mechanism of brucellosis remains unclear. In this study, we performed a global proteomic analysis of the whole protein of *Brucella abortus* to create a PTM-omic atlas that includes multiple types of PTMs. Studying PTMs of *Brucella* proteins and the involved pathways is of great worth for revealing the pathogenic mechanism of brucellosis and developing new drugs for its treatment.

## Materials and Methods

### Bacterial Culture and Protein Extraction

Virulent *B. abortus* 2308 was obtained from Tecon Biological Co., Ltd. (Urumqi, China) and cultured in tryptone soy agar (TSA). The colonies were cultured in TSA medium at 37°C in a shaking incubator. Before performing analysis of PTMs, we extracted the whole proteins of *Brucella* in exponential stage and stationary growth stage in three separate preliminary experiments, and found that the modification level of proteins in exponential stage is higher than that in stationary growth stage through detecting by western blot. Besides, *Brucella* has better growth activity (Yang et al., 2021) and invasiveness (Rossetti et al., 2009) in exponential stage, so we finally chose *Brucella* in exponential stage as research object in this study (Detailed data are listed in supplemental Supplementary Figure S1). The bacteria solution was sonicated three times on ice using a high intensity ultrasonic processor in lysis buffer (8 M urea, 1% protease Inhibitor Cocktail) (protease Inhibitor Cocktail, Beyotime, China, P1005). The remaining cell debris was removed by centrifugation at 12,000 g at 4°C for 10 min (Wang et al., 2019). The supernatant was collected, and the protein concentration was determined using a BCA kit (Beyotime, China, P0012) according to the manufacturer’s instructions.

### Trypsin Digestion

For digestion, the protein solution was reduced with 5 mM dithiothreitol for 30 min at 56 °C and alkylated with 11 mM iodoacetamide for 15 min at 25°C in the dark. The protein sample was then diluted by adding 100 mM TEAB (tetraethyl-ammonium bromide) to a urea concentration of less than 2 M. Finally, trypsin was added at a 1:50 trypsin-to-protein mass ratio for the first digestion overnight and 1:100 trypsin-to-protein mass ratio for a second 4 h digestion.

### Pan Antibody-Based PTM Enrichment (for Lysine 2-Hydroxyisobutyrylation, Lysine Succinylation, Lysine Crotonylation, Lysine Acetylation, and Lysine Malonylation)

To enrich lysine 2-hydroxyisobutyrylation, lysine succinylation, lysine crotonylation, lysine acetylation, and lysine malonylation modified peptides, tryptic peptides dissolved in NETN buffer (100 mM NaCl, 1 mM EDTA, 50 mM Tris-HCl, 0.5% NP-40, pH 8.0) were incubated with pre-washed antibody beads (PTM-804 for 2-hydroxyisobutyrylation, PTM-409 for succinylation, PTM-503 for crotonylation, PTM-104 for acetylation, and PTM-901 for malonylation, PTM Bio) at 4°C overnight with gentle shaking. Then the beads were washed four times with NETN buffer and twice with H_2_O. The bound peptides were eluted from the beads with 0.1% trifluoroacetic acid. Finally, the eluted fractions were combined and vacuum-dried. For LC-MS/MS analysis, the resulting peptides were desalted with C18 ZipTips (Millipore) according to the manufacturer’s instructions.

### LC-MS/MS Analysis

The tryptic peptides were dissolved in 0.1% formic acid (solvent A) and directly loaded onto a homemade reversed-phase analytical column (15-cm length, 75-μm internal diameter). The gradient comprised an increase from 6 to 23% solvent B (0.1% formic acid in 98% acetonitrile) over 26 min, then 23–35% over 8 min, and 35–80% over 3 min. Elution was completed by holding at 80% for the last 3 min. The procedure was performed at a constant flow rate of 400 nL/min using an EASY-nLC 1000 ultra-performance liquid chromatography (UPLC) system.

The peptides were subjected to NSI source followed by MS/MS in Q ExactiveTM Plus (Thermo Fisher Scientific, Waltham, United States) coupled online to the UPLC. The applied electrospray voltage was 2.0 kV. The m/z scan range was 350 to 1,800 for a full scan, and intact peptides were detected in the Orbitrap at a resolution of 70,000. Peptides were then selected for MS/MS using a NCE setting of 28, and the fragments were detected in the Orbitrap at a resolution of 17,500. We followed a data-dependent procedure that alternated between one MS scan and 20 MS/MS scans with a 15.0 s dynamic exclusion. Automatic gain control was set at 5E4. The fixed first mass was set to 100 m/z.

### Expression and Purification of BspF *in vitro*

The BspF gene was amplifified by PCR and cloned into pET-GST (Bioon, Shenzhen, China, zt208) to produce GST-tagged fusion proteins with a PreScission protease cleavage site between GST and the target proteins. The proteins were expressed in *E. coli* strain Rosetta and induced by 0.2 mM isopropyl-b-D-thiogalactopyranoside (IPTG) when the cell density reached an OD_600nm_ of 0.8. After growth at 16°C for 12 h, the cells were harvested, re-suspended in lysis buffer (1×PBS, 2 mM DTT and 1 mM PMSF) and lysed by sonication. The cell lysate was centrifuged at 20,000 g for 45 min at 4°C to remove cell debris. The supernatant was applied onto a self-packaged GST-affifinity column (2 ml glutathione Sepharose 4B; GE Healthcare, United States) and contaminant proteins were removed with wash buffer (lysis buffer plus 200 mM NaCl). The fusion protein was then digested with PreScission protease at 4°C overnight. The protein was eluted with lysis buffer. The eluant was concentrated and further purifified using a Superdex-200 (GE Healthcare, United States) column equilibrated with a buffer containing 10 mM Tris–HCl pH 7.8, 500 mM NaCl, and 5 mM DTT. Refer to Supplementary Figure S2.

### Cell Culture, Plasmid Transfection, and Overexpression of RicA

HEK-293T and HeLa cells were cultured in Dulbecco’s minimal essential medium containing 10% fetal bovine serum (FBS, Gemin, United States, A73D00E) at 37°C and 5% CO_2_. Plated cells were cultured in 10 ml of 10% growth medium for 1 day before transfection, until they reached 70–90% confluency. Lipofectamine 2000 reagent and the plasmids were diluted in Opti-MEM medium (Gibco, Thermo Fisher Scientific, United States, 2085119) and incubated for 10 min. The diluted plasmids and Lipofectamine 2000 were gently mixed, incubated for 30 min at room temperature, and then added to the cells. Six hours after transfection, the growth medium was removed from the cells and replaced with 10 ml of 2% FBS maintenance medium. Test plates were transfected with 16 μg of HA-RicA plasmid (constructed by our lab), and the control plates were transfected with 16 μg of pCMV-HA (Bioon, Shenzhe, China, zt296) plasmid. The cells were collected 30 h after transfection. Protein expression level was assessed using western blotting.

### Western Blot

Cell lysates were harvested at 30 h, and the protein concentration was determined. Equivalent quantities of cell lysates were denatured in 5X loading buffer and boiled at 100°C for 10 min. Equal amounts of proteins were separated by 10% SDS-PAGE (EpiZyme, China, PG112) and transferred to polyvinylidene fluoride (PVDF) membranes (Merck Millipore, United States, ISEQ00010). The membranes were then washed in Tris-buffered saline with Tween 20 (TBST) and blocked in TBST containing 5% skimmed milk for 2 h at 25°C. Membranes were incubated overnight at 4 °C with antibodies for detecting Cr (PTM Bio, China, PTM-545RM), HA (Beyotime, China, AF0039) and β-actin (Beyotime, China, AF5001), followed by incubation with HRP-conjugated secondary antibody (Beyotime, China, A0192) at 25°C for 2 h. Signals were detected with Clarity ECL (Enhanced Chemiluminescence) reagents (Beyotime, China, P0018FS).

### Database Search

The MS/MS data were processed using the MaxQuant search engine (v.1.5.2.8). Tandem mass spectra were searched against the SwissProt database concatenated with the reverse decoy database. The mass spectrometry proteomics data have been deposited to the ProteomeXchange Consortium via the PRIDE partner repository with the dataset identifier PXD030621. Trypsin was specified as a cleavage enzyme, allowing up to two missing cleavages. The mass tolerance for precursor ions was set as 20 ppm in the first search and 5 ppm in the main search, and the mass tolerance for fragment ions was set as 0.02 Da. Carbamidomethyl on cysteine was specified as a fixed modification, and oxidation on methionine was specified as a variable modification. FDR (false discovery rate) was adjusted to <1%, and the minimum score for peptides was set at > 40.

### GO and KEGG Pathway Annotation

The GO annotation proteome was derived from the UniProt-GOA database (http://www.ebi.ac.uk/GOA/). First, the protein identity (ID) was converted to UniProt ID and then mapped to GO IDs by protein ID. If some identified proteins were not annotated by the UniProtGOA database, the InterProScan software was used to annotate the GO function of the protein based on the protein sequence alignment method. Subsequently, proteins were classified by Gene Ontology annotation based on three categories: biological process, cellular component, and molecular function. The KEGG (Kyoto Encyclopedia of Genes and Genomes) database was used to annotate protein pathways. First, the KEGG online service tool KAAS (KEGG Automatic Annotation Server) was used to annotate the proteins’ KEGG database description. The annotation results were mapped to the pathway database using the online service tool KEGG mapper.

### Subcellular Localization

The subcellular localization prediction software wolfpsort was used to predict subcellular localization. Wolfpsort is an updated version of PSORT/PSORT II for the prediction of eukaryotic sequences. For prokaryotic species, the subcellular localization prediction software CELLO was used.

### Enrichment of GO and Pathways

Proteins were classified by GO annotation into three categories: biological process, cellular compartment, and molecular function. For each category, a two-tailed Fisher’s exact test was employed to test the significance of enrichment of the identified proteins against all proteins in the species database. The KEGG enriched pathways were similarly identified. Pathways and GO-terms with a corrected p-value < 0.05 were considered significant. Significant pathways were classified into hierarchical categories according to the KEGG website.

### Protein-Protein Interaction Network Analysis

All identified protein database accessions or sequences were searched against the STRING database version 10.5 for protein-protein interactions. Only interactions between the proteins belonging to the searched data set were selected, thereby excluding external candidates. STRING defines a metric called “confidence score” to define interaction confidence; we identified all interactions that had a high confidence score (>0.7). The interaction network from STRING was visualized in the R package networkD3.

### Statistical Analysis

All data are analyzed with GraphPad Prism version 8 (GraphPad Software; San Diego, CA) and expressed as means ± SEM. Statistical significance was calculated using two-tailed Student’s t test, unless stated otherwise. p-values of <0.05 (typically ≤0.05) were regarded as statistically significant. NS stands for not statistically significant.

## Results

### Proteomic Profile and Acylation Atlas of *B. abortus*

**FIGURE 1:**
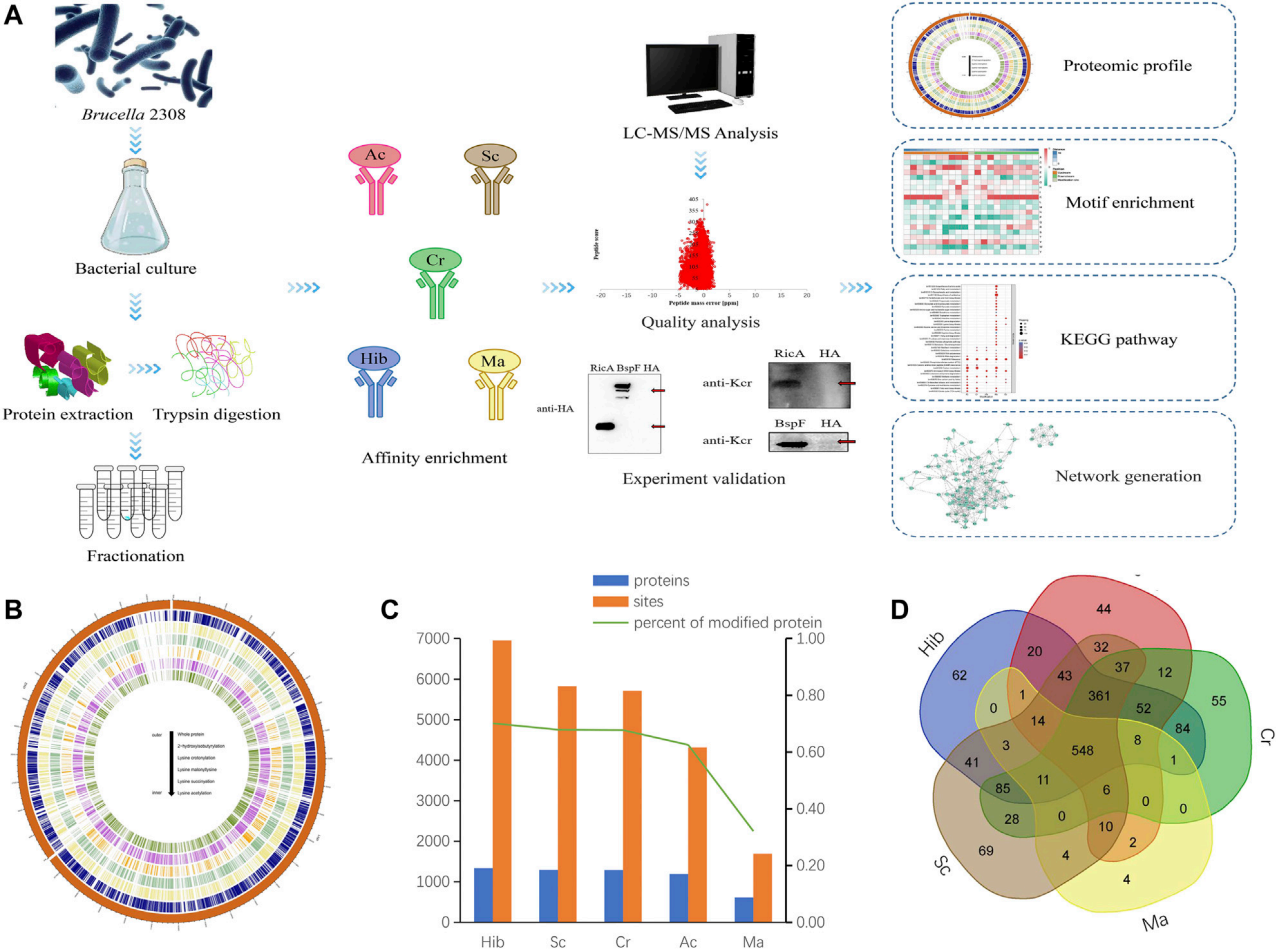
Acylation Modifications of *B. abortus* 2308. Flowchart of the proteomic procedures for protein and modification site identification (A). Circos plot showing the abundance configurations of five modification omics in chromosome distribution **(B)**. Histograms of proteomes and five PTM-omics of *B. abortus* 2308. The number of identified proteins and modification sites are included. The number of modified proteins (blue), the number of modification sites (orange) **(C)**. Venn diagram showing overlap among five lysine modified proteins in *B. abortus* 2308. Ellipses in blue, red, green, yellow and brown represent the numbers of proteins modified by lysine 2-hydroxyisobutyrylation, acetylation, crotonylation, malonyllysine and succinylation identified in *B. abortus* 2308, respectively. Overlap regions represent the number of proteins with all modifications in *B. abortus* 2308 **(D)**.

Combined the growth activity, invasiveness and modification level of *Brucella* in two stages, the *Brucella* in exponential stage was chosen in our study (Supplementary Figure S1). To obtain an overall view of lysine modification levels and patterns, we performed a global proteomic analysis of *Brucella* using tryptic digestion, antibody affinity enrichment, and high-resolution liquid chromatography-tandem mass spectroscopy (LC-MS/MS). lysine 2-hydroxyisobutyrylation (Hib), lysine succinylation (Sc), lysine crotonylation (Cr), lysine acetylation (Ac), and lysine malonylation (Ma) were assessed (Figure 1A). The circos plot shows the abundance configurations of the proteome and five lysine PTM-omics for proteins isolated from *B. abortus* 2308. All five modification-types have similar location distributions in chromosome I (2,121,359 bp), whereas in chromosome II (1,156,948 bp), lysine acetylation and lysine succinylation were more prominent (Figure 1B).

Across the five lysine PTM-omics, we identified 1,904 modified proteins in the *B. abortus* proteome. The most frequent type was Hib, while Ma was least frequent. In the identified proteins the distribution of modification sites across proteins were as follows: Hib 6,953 sites and 1,336 proteins; Sc,5,825 sites and 1,293 proteins; Cr, 5,709 sites and 1,290 proteins; Ac, 4,317 sites and 1,191 proteins; and Ma, 1,693 sites and 612 proteins (Figure 1C). The number of malonylated proteins was considerably lower than that of the other four PTM-omics. These five PTM-omics shared 548 proteins, and most proteins (≥96%) underwent more than one modification. Approximately 4% of proteins had only one type of PTMs. Specifically, 69, 62, 44, 55, and 4 proteins underwent only Sc, Hib, Ac, Cr, and Ma, respectively (Figure 1D). These proteins were involved in energy metabolism and protein synthesis. By statistical analysis of amino acid sequence before and after all Hib, Sc, Cr, Ac and Ma in samples, we calculated the trend of amino acid sequence in the region of Hib, Sc, Cr, Ac and Ma sites. Such analysis can reveal the sequence characteristics of the modified site and thus infer or identify the enzyme associated with the modification (Supplementary Figure S3).

### Validation of Mass Spectrometry Data and Verification of Acylated Proteins

To validate the mass spectrometry data and to ensure that sample preparation reached standard conditions, the mass error and peptide length of the identified peptides were examined. As shown in Supplementary Figure S4, the mass error is less than 10 ppm, and the precision satisfies the bioinformatics analysis. For peptide length (Supplementary Figure S5), the majority of the distribution was between seven and twenty amino acids, which is in accordance with the characteristic length of tryptic peptides. MS/MS information related to these PTM peptides was deposited in the iProX database with the accession number.

**FIGURE 2:**
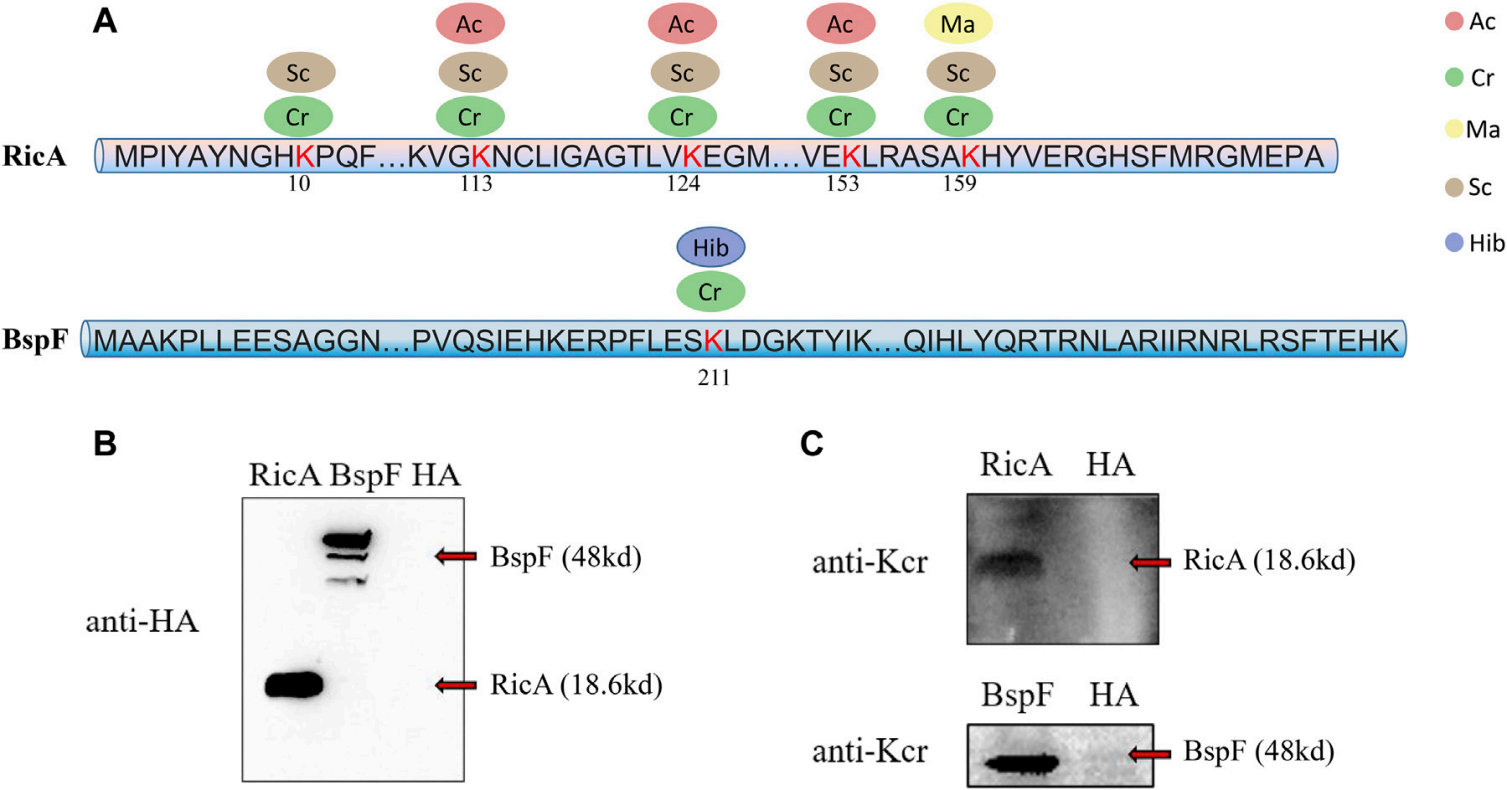
Overview of five lysine modified sites identified on RicA and BspF of *B. abortus* 2308 and Western blot verification. Five lysine modified sites of RicA and BspA are shown as **(A)**. Ellipses in blue, red, green, yellow and brown represent the numbers of sites modified by lysine 2-hydroxyisobutyrylation, acetylation, crotonylation, malonyllysine and succinylation. Numbers in the middle of the sequences indicate the amino acid position on histon. RicA and BspF were expressed normally verified by Western Blot with anti-HA antibody in **(B)**. Western blot analysis of 20 μg of RicA and BspF with anti-crotonyllysine antibodies. RicA and BspF were detected in the expected place where is 19.25 and 47.08 kd respectively as shown in **(C)**.

To determine the reliability of the LC-MS/MS data, we conducted a verification experiment using an antibody against the crotonyl group. We selected two effector proteins, RicA and BspF, for validation experiments *in vivo* and *in vitro*, respectively. According to the LC-MS/MS data, RicA (BAB1-1279) underwent four types of PTMs (Cr, Ma, Sc, and Ac), and BspF (BAB1-1948) underwent two types of PTMs (Hib and Cr). Both were crotonylated, but RicA had five Cr sites, whereas BspF had only one (Figure 2A). Therefore, we investigated the Cr of these two proteins. As expected, RicA and BspF were detected with the Cr antibody *in vivo* and *in vitro*, respectively (Figures 2B,C). These results were consistent with the mass spectroscopy data.

### Subcellular Location Landscapes of Protein Acylation PTMs in *Brucella*

**FIGURE 3:**
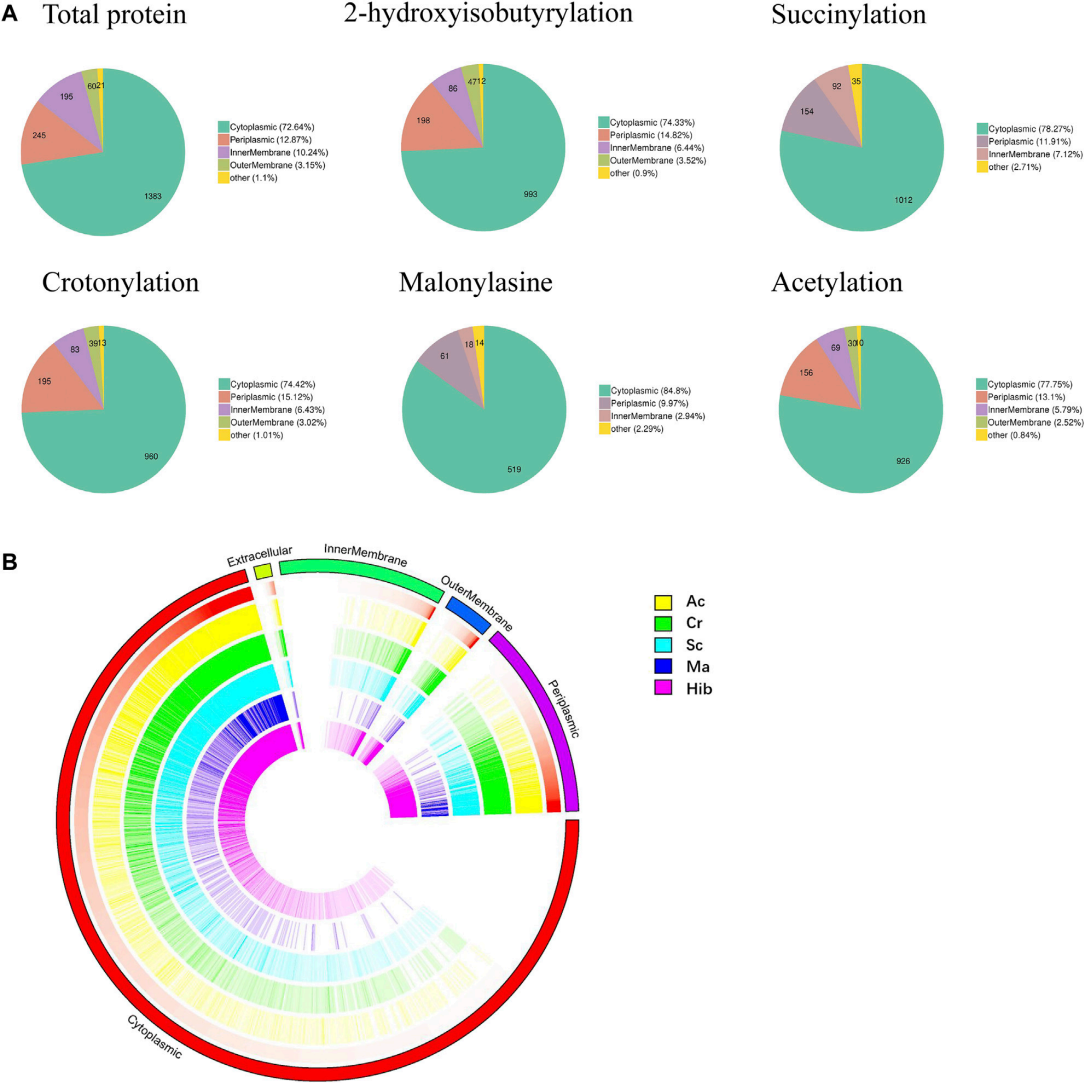
Subcellular localization analysis of lysine modified proteins. Pie charts showing subcellular localization of each type of PTM, more than 70% proteins located in cytoplasmic **(A)**. Visual presentation of lysine modified proteins based on subcellular location in *B. abortus* 2308 **(B)**. The outermost circle of the circos plot means different subcellular localization, cytoplasmic (red), periplasmic (purple), outer membrane (dark blue), inner membrane (green), and extracellular (dull yellow). Five circles from the outside represents different modified sites (Hib, Cr, Sc, Ma and Ac), respectively. A deeper color represents higher enrichment of the modification. Note that the height of each bar reflects the subcellular organelle abundance of each protein or PTM site, i.e., longer bars represent a greater degree of abundance.

Subcellular localization of modified proteins in *B. abortus* 2308 provides clues about the possible functions of the modified proteins. The results (Figure 3A) showed that the largest proportion of modified proteins was assigned to cytoplasmic (Hib, 74%; Sc, 78%; Cr, 74%; Ac, 78%; and Ma, 85%), followed by periplasmic (Hib, 15%; Sc, 12%; Cr, 15%; Ac, 13%; and Ma, 10%) and membrane (Hib, 10%; Sc, 7%; Cr, 10%; Ac, 8%; and Ma, 3%) compartments. Overview of the subcellular localization of modified proteins is shown in Figure 3B. All five types of modification had similar subcellular location distributions. Interestingly, neither Sc nor Ma affected the proteins identified in the outer membrane, which indicates that these two modifications do not occur in outer membrane proteins. For example, lptD is located in the extracellular membrane and is involved in the synthesis of *Brucella* Lipopolysaccharide (LPS), but neither Sc nor Ma was detected.

### Intracellular Survival-Related Metabolic Pathways Were Enriched in Acylation PTMs

**FIGURE 4:**
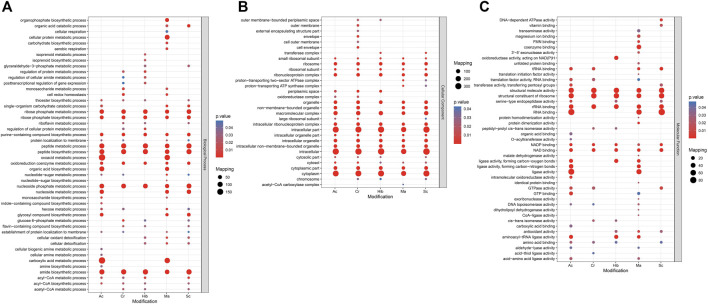
GO (Gene Ontology) enrichment bubble plot of proteins corresponding to modification sites in three categories [**(A)** Biological Process; **(B)** Cellular Component; **(C)** Molecular Function]. The GO with a corrected p-value < 0.05 is considered significant.

Gene ontology (GO) analysis and Kyoto encyclopedia of genes and genomes (KEGG) pathway enrichment analysis were used to understand the biological significance of the five types of modifications. Statistical distribution charts of modified proteins under GO categories are shown in Figure 4. Cr, Hib, and Sc were similarly distributed across biological processes, while distribution of Ac and Ma were similar. All five modifications occurred on proteins mainly located in the ribose phosphate metabolic process, ribose phosphate biosynthetic process, purine-containing compound biosynthetic process, peptide metabolic process, peptide biosynthetic process, oxidoreduction coenzyme metabolic process, nucleoside phosphate metabolic process, and amide biosynthetic process. Among the five modifications, Ma was more widely distributed, and Ac was more concentrated. The flavin-containing compound biosynthetic process, glucose-6-phosphate metabolic process, and riboflavin metabolic process were associated with Cr, Hib, and Sc. Ac and Ma were mainly involved in carboxylic acid metabolic processes, oxoacid metabolic processes, and organic acid biosynthetic processes. As for cellular components, all five modifications were mainly enriched in intracellular part, intracellular, and cytoplasmic part. Cr was the most widely distributed in the cellular components, and only Cr participated in the outer membrane, outer cell membrane, envelope, and cell envelope; Hib did not participate in non-membrane-bound organelles, and Ma was absent in the cytosolic part. Concerning molecular functions, all five modifications were closely related to catalytic activity. Cr, Hib, Sc, and Ac were associated with transport activity, but Ma was associated with structural molecular activity. In addition, all five modifications are ubiquitous in energy generation and exchange as well as in replication, recombination, and repair processes. In particular, malonylated proteins was more prominent in the processes of translation and the structure of the ribosome, but their enrichment in processes of cell cycle regulation, cell division, and chromosome segmentation were markedly lower compared to that of the other four modifications. Consequently, these five modifiers appear to collectively perform their respective functions to regulate the intracellular survival of *Brucella*.

**FIGURE 5:**
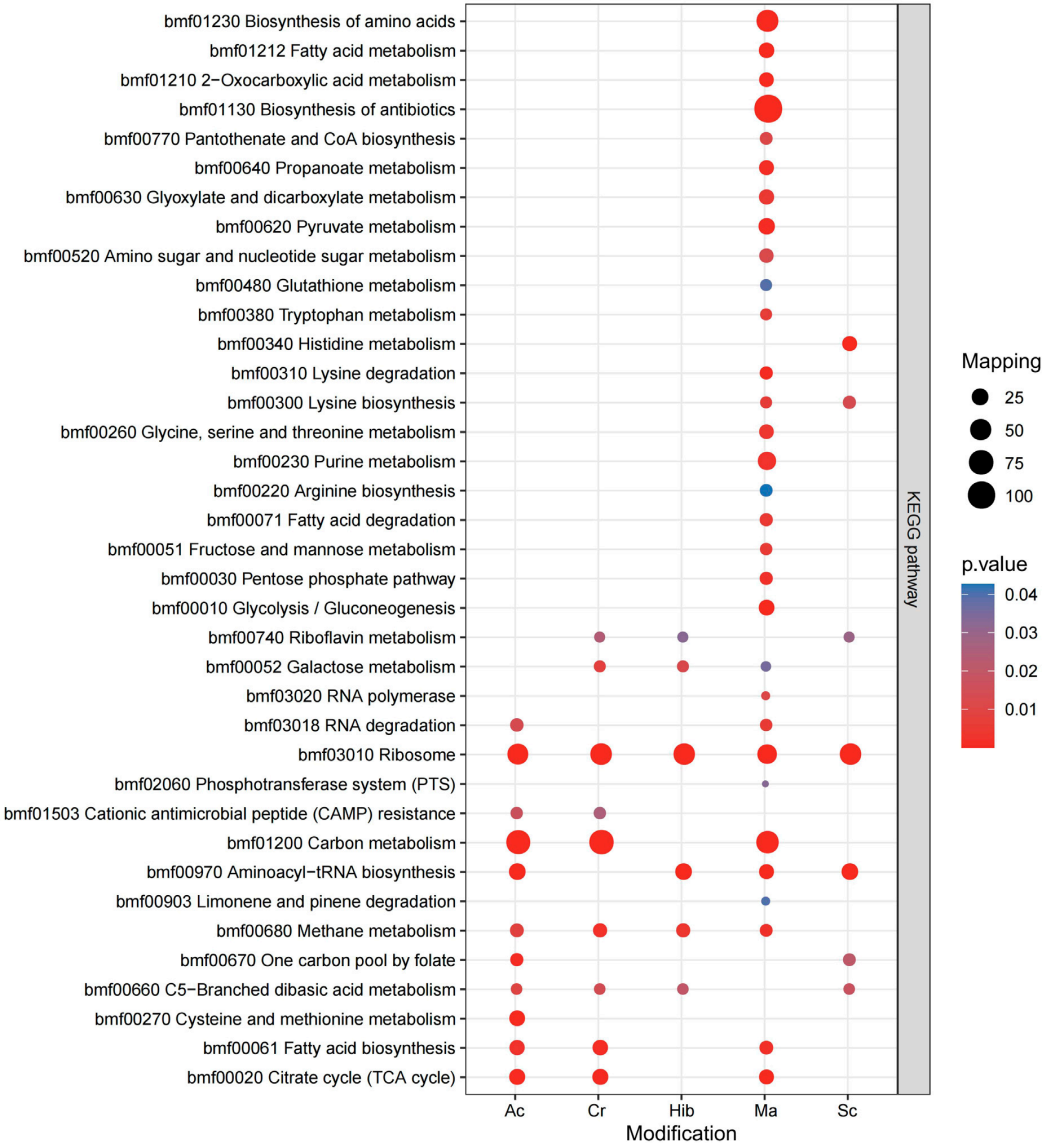
KEGG pathway enrichment bubble plot of proteins corresponding to modification sites.

KEGG is an information network that connects known molecular interactions, such as metabolic pathways, complexes, and biochemical reactions. In *Brucella*, these five modifications were observed for different functions, with proteins enriched in different pathways. The KEGG pathway enrichment bubble plots of proteins corresponding to the modification sites are shown in Figure 5. Sc mainly regulated the synthesis of lysine, while Hib was involved in glucose metabolism pathways, which was in agreement with previous research showing that Hib affects the glycolysis process in yeast (Huang et al., 2017). Ac mainly affected carbon and fatty acid metabolism in *Brucella*, whereas Cr was involved in carbon metabolism, fatty acid metabolism, and the TCA cycle, and many crotonylated proteins were related to ribosomes, which is consistent with a previous study showing that the transcription and replication of genes are closely related to lysine Cr (Wei et al., 2017a), (Wei et al., 2017b). Ma was mainly enriched in KEGG pathways related to the TCA cycle, antibiotic synthesis, and amino acid synthesis.

### Highly Acylated Virulence Proteins in *Brucella*

Through biological analysis of the PTM-omics data, we found that a large number of proteins involved in virulence were modified. To study and discuss the effect of modification on *Brucella*, we screened a few classes of proteins that are important during the growth, reproduction, invasion, and infection of *Brucella*, including virulence factors, stress particles, and essential genes, which may help *Brucella* to survive intracellularly in the host and cause persistent infection (Tables 1, 2). In Table 1, we list the proteins associated with immune evasion (lpxC, lpxD, fabZ, lpxA, lpxB, kdsA, htrB, kdsB, acpXL, lpxE, wboA, pgm, wbpZ, manAoAg, manCoAg, pmm, wbkA, gmd, per, wzm, wzt, wbkB, wbkC, lpsA, lpsB/lpcC, wbdA, wbpL, manBcore, manCcore, lpxK, and waaA/kdtA), the modification of these proteins may enhance the ability of *Brucella* to escape immune phagocytosis of host cells. About intracellular survival (CbetaG), regulation (bvrR and bvrS), and T4SS structural proteins (VirB1–11), we thus hypothesized that the modification would improve the ability of *Brucella* to help the bacteria survive intracellularly in the host. Notably, we observed that most of these virulence factors were associated with PTMs. In addition, 10 of 15 known effectors of T4SS (RicA, VceA, VceC, BPE043, BtpA, BspB, BspC, BspE, BspF, and SepA) underwent various modifications. VceA can suppress autophagy in the process of infection, thereby to escape the immune killing system of the host, which is conducive to the intracellular survival of *Brucella*. VceC can promote autophagy in the process of infection, which is unhelpful for escaping the immune killing system of the host. In Table 2, VceC is modified by all five of these modifications but VceA is not modified by Ac and Ma, we hypothesized that these two modifications may regulate the autophagy process and thus affect the intracellular survival of *Brucella* (Zhang et al., 2019b). Moreover, essential genes (for example, SerS, FusA, and RpsL), one of the 14 membrane proteins of *Brucella* (Omp31-1), and regulatory proteins (sodC), which can help bacteria escape the killing effects of phagocytes, are modified by all five of these modifications (Table 2). Consequently, it is likely that the five modifications play a crucial role in virulence and these modified sites can potentially serve as therapeutic targets for brucellosis.

**TABLE 1:** Modification of classical virulence factors in *Brucella*.

Virulence factors	Related genes	*B. melitensis* biovar abortus 2308		Protein accession	PTMs				
		Chromosome I NC_007618 (2121359 bp)	Chromosome II NC_007624 (1156948 bp)		Hib	Sc	Ac	Cr	Ma
Immune evasion									
LPS	lpxC	BAB1_1443		Q2YLZ2	1	3	3	2	0
	lpxD	BAB1_1175		Q2YRQ3	2	2	3	1	2
	fabZ	BAB1_1174		Q2YRQ4	2	3	3	3	1
	lpxA	BAB1_1173		Q2YRQ5	3	0	2	1	0
	lpxB	BAB1_1171		Q2YMP4	1	1	0	0	0
	kdsA	BAB1_1156		Q2YPU9	8	8	6	9	2
	htrB	BAB1_0870		Q2YNI7	0	2	0	0	0
	kdsB	BAB1_0035		Q2YPQ5	6	5	2	4	5
	acpXL	BAB1_0874		Q2YNI3	4	4	5	3	3
	lpxE	BAB1_0761		×					
	wboA	BAB1_0999		Q2YQ89	5	14	4	4	5
	pgm	BAB1_0055		Q2YPS4	19	18	15	14	9
	wbpZ			×					
	manAoAg	BAB1_0563		×					
	manCoAg	BAB1_0561		Q2YMQ9	4	7	5	4	2
	pmm	BAB1_0560		×					
	wbkA	BAB1_0553		Q2YMN5	2	1	0	0	0
	gmd	BAB1_0545		Q2YMP3	8	9	8	7	7
	per	BAB1_0544		Q2YMP4	5	5	8	3	3
	wzm	BAB1_0543		×					
	wzt	BAB1_0542		Q2YMP6	5	2	5	3	0
	wbkB	BAB1_0541		×					
	wbkC	BAB1_0540		Q2YML4	3	2	2	2	0
	lpsA	BAB1_0639		×					
	lpsB/lpcC	BAB1_1522		Q2YRN9	0	1	2	1	0
	wbdA	BAB1_1000		Q2YQ88	7	8	5	5	1
	wbpL	BAB1_0535		Q2YML9	0	2	1	1	0
	manBcore		BAB2_0855	Q2YK25	5	7	3	2	1
	manCcore		BAB2_0856	Q2YK24	3	4	2	2	1
	lpxK		BAB2_0210	×					
	waaA/kdtA		BAB2_0209	Q2YIH5	3	1	1	2	0
Intracellular survival									
CbetaG	cgs	BAB1_0108		Q2YNV7	10	13	5	11	0
Regulation									
BvrRS	bvrR	BAB1_2092		Q2YQY4	7	6	5	6	2
	bvrS	BAB1_2093		Q2YQY3	2	2	1	1	1
Secretion system									
Type IV secretion system	virB1		BAB2_0068						
	virB2		BAB2_0067						
	virB3		BAB2_0066						
	virB4		BAB2_0065	Q2YIT8	0	0	0	1	0
	virB5		BAB2_0064	Q2YJ75	0	0	0	1	0
	virB6		BAB2_0063						
	virB7		BAB2_0062						
	virB8		BAB2_0061	Q2YJ78	4	3	0	4	1
	virB9		BAB2_0060	Q2YJ79	4	2	3	5	0
	virB10		BAB2_0059	Q2YJ81	4	1	1	2	0
	virB11		BAB2_0058	Q2YJ82	2	3	2	1	0

**TABLE 2:** Modification of effectors, essential genes and regulatory proteins in *Brucella*.

Virulence factors	Related genes	*B. melitensis* biovar abortus 2308		Protein accession	PTMs				
		Chromosome I NC_007618 (2121359 bp)	Chromosome II NC_007624 (1156948 bp)		LP2o	LPSc	LPAc	LPCr	LPMa
Effectors									
	RicA	BAB1_1279		Q2YQG1	4	5	3	5	1
	VceA	BAB1_1652		Q2YRI6	5	1	0	3	0
	VceC	BAB1_1058		Q2YQ34	2	2	1	2	2
	BPE043	BAB1_1043		Q2YQ47	23	19	10	17	4
	BtpA	BAB1_0279		Q2YPC4	1	0	0	0	0
	BspB	BAB1_0712		Q2YN43	1	0	0	0	0
	BspC	BAB1_0847		Q2YNH3	8	4	5	7	1
	BspE	BAB1_1671		Q2YRH9	4	3	4	4	1
	BspF	BAB1_1948		Q2YLT8	1	0	0	1	0
	SepA	BAB1_1492		Q2YS16	2	0	0	0	0
Essential genes									
	FtsZ	BAB1_1444		Q2YLZ1	7	5	3	4	0
	SerS	BAB1_0904		Q2YNM6	11	14	7	11	5
	FusA	BAB1_1258		Q2YM00	25	23	17	23	14
	RpsL	BAB1_1260		Q2YLZ8	6	3	2	2	2
Membrane proteins									
	Omp31-1	BAB1_1639		Q2YQC2	6	4	5	6	1
Regulatory proteins									
	sodC		BAB2_0535	Q2YKV9	10	9	10	10	4

### Complex Protein-Protein Interaction of Acylated Proteins

**FIGURE 6:**
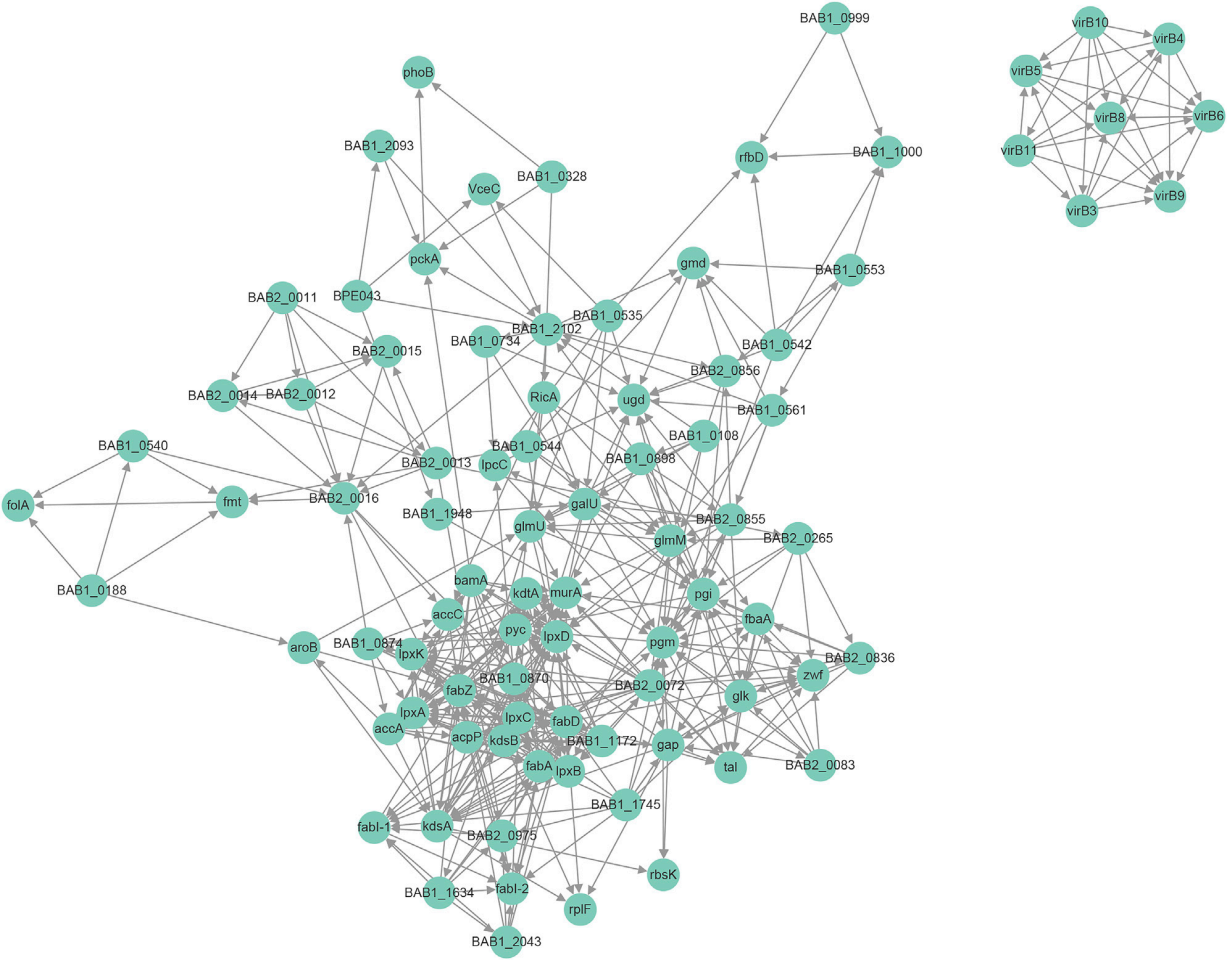
Protein-protein interaction network for modified virulence raleated proteins corresponding to modification sites.

Protein interaction networks are composed of individual proteins that interact with each other to participate in gene expression regulation, cell cycle regulation, biological signal transmission, energy and substance metabolism, and other processes. Among the 1904 modified proteins in *Brucella*, we screened 48 modified virulence-related proteins and constructed an interaction network (Figure 6). Here, we found that the LPS-associated proteins were mainly modified by Hib and Sc, with lesser amounts of Cr and Ac; proteins modified by Ma were minimal. The LPS-associated proteins, including pgm, wbdA, manBcore, wboA, wbkC, and gmd were mainly Hib and Sc, but they also exhibited Cr and Ac. However, wbkC was not modified by Ma. The modification types of the effectors and essential genes were more diverse, with prominent Hib, Sc, and Cr, and lesser amounts of Ac and Ma. Modification of RicA, BPE043, BspC, SerS, and FusA were consistent with this trend; however, the essential genes FtsZ were not modified by Ma. The physiological interaction between these modified proteins may contribute to their synergistic effect in *B. abortus* 2308.

## Discussion

The extensive damage caused by *Brucella* to human health and the livestock industry has prompted increased research activities. Although previous studies have explored the pathogenesis of brucellosis, how *Brucella* achieve intracellular survival in their host cells and the molecular mechanisms of virulence remains unclear (Celli, 2019), (Gorvel and Moreno, 2002). The pathogenicity of *Brucella* species is mainly defined by specific virulence factors and effector proteins, which are crucial for their survival. Precise control of proteins is essential for the functioning of the organism. Among the different regulatory processes, reversible PTM is an excellent mechanism for controlling protein function. Therefore, analyzing the *Brucella* proteome and PTMs may contribute to a more comprehensive understanding of its adaptive mechanism within host cells.

In this study, we identified a large dataset of 1904 proteins in *Brucella* with five different PTMs, namely Hib, Sc, Cr, Ac, and Ma. From the proteome, we found that many virulence factors of *Brucella* and T4SS effector proteins had undergone PTMs. For instance, VirB9, VirB10, and VirB11 are structural proteins of T4SS, all of which underwent simultaneous Hib, Ac, Cr, and Sc. VirB10 interacts with VirB8 via the beta1-strand (Sharifahmadian et al., 2017), which means that the modification of these three proteins may affect the secretion of effectors. When acting sequentially, the modification of effectors may result in changes to the cellular functions of *Brucella*, and this series of processes may provide clues to the pathogenesis of brucellosis. VceA and VceC are two effectors of T4SS (de Jong et al., 2008), and both of them underwent more than one type of PTM, which suggests that the survival of *Brucella* in the host may be related to modification. In the current study, BspF was detected with one Cr site. BspF is an effector member of T4SS that can inhibit host cell protein secretion and promote *Brucella* intracellular growth and persistence (Myeni et al., 2013). Moreover, our previous study indicated that BspF can change the intracellular Cr level to promote the survival of *Brucella* (Zhu et al., 2020), which provided insights into the effect of modification on protein function. VirJ is a *Brucella* virulence factor involved in the T4SS secreted substrates, which undergoes two different types of PTMs, namely Hib and Cr. Therefore, we suggest that the PTM of proteins related to T4SS are involved in regulating their interaction with host cells.

LPS is another major virulence factor of *Brucella* that plays an important role in the invasion of host cells (Pei and Ficht, 2011). We identified several proteins associated with LPS synthesis. Among them, phosphoglucomutase (BAB1_0544) and DegT/DnrJ/EryC1/StrS aminotransferase underwent all five types of PTMs. Both of these proteins are involved in O-chain synthesis, which is the main factor that determines the virulence of *Brucella*. Cgs (BAB1_0108) is a virulence factor that interacts with lipid rafts and contributes to pathogen survival, and is important not only for evasion of lysosome degradation but also for the ability of bacteria to reach the nonhostile endoplasmic reticulum replication niche. Interestingly, Cgs was repeatedly modified by Hib, Sc, Ac, and Cr, but not by Ma. Sc occurred at 13 sites in Cgs. In addition, lptD is involved in the synthesis of *Brucella* LPS. In this study, we found that lptD had undergone Hib, Ac, and Cr, but, as with Cgs, no site was modified by Ma. Since the proteins involved in LPS and intracellular survival are likely to be modified through Hib, Ac, Cr, and Sc, but not Ma, these specific modifications may have roles in cell invasion and intracellular survival of *Brucella*.

Previous studies have shown that SOD (superoxide dismutase) is part of an antioxidant defense system that protects cells from the toxic effects of oxygen-mediated superoxide ion conversion to hydrogen peroxide (Gee et al., 2005). Cu/Zn SOD underwent all five of the modification types evaluated in this study. Studying the modification of Cu/Zn SOD will provide new ideas for future research on functions and mechanisms and has important implications for understanding how *Brucella* avoids being killed by host cells. FtsZ is a GTP enzyme necessary for cell division in prokaryotic cells and is considered to be an essential gene for *Brucella*. SerS and FusA are genes involved in the synthesis of aminoacyl tRNA synthase and the synthesis of translation initiation factor, respectively, while RpsL is a gene encoding ribosomal protein small subunit S12, as well as a virulence gene for *Brucella*, and is required for brucellosis candidates. SerS, FusA, and RpsL underwent all five types of PTMs, but Ma did not occur in FtsZ. Omp31-1, one of fourteen outer membrane proteins, is conserved in virulent strains that are pathogenic in humans, including *B. abortus*, *B. melitensis*, and *B. suis*, but not in *B. ovis*, a species that is not pathogenic in humans. In our study, there were six, four, five, and six sites modified by Hib, Sc, Ac, and Cr, respectively, but only one site had Ma. These essential proteins and Omp31-1 were widely modified by the five types of PTMs, indicating that modification may affect the intracellular survival and virulence of *B. abortus* 2308.

Based on our proteomic data, we comprehensively screened and identified essential genes, cell adsorption and invasion-related proteins, and those related to physical, chemical, and biological factor tolerance in *B. abortus* 2308. These studies provide the first atlas of PTM in *Brucella*. We will further design experiments concerning the effect of modified proteins on the survival of *Brucella* and explore whether PTMs affect their function, invasion, and infection ability. Therefore, our data not only expanded the *Brucella* spp. protein PTM dataset but also laid a foundation for functional investigation of proteins with these five PTMs during their reproduction and survival.

## Data Availability

The mass spectrometry proteomics data have been deposited to the ProteomeXchange Consortium via the PRIDE partner repository with the dataset identifier PXD030621.

## Supplementary Material

Supplementary Figure S1 Western blot analysis of 20 μg of totol protein quantify with BCA (Beyotime, China, P0012) with anti-acetyllysine and anti-crotonyllysine antibody. Lane1 presents totol protein of *B. abortus* 2308 in stationary growth stage, lane 2 presents totol protein of *B. abortus* 2308 in exponential stage.

Supplementary Figure S2 SDS-PAGE of samples during BspF purification. None induction (NI), induction (I), supernatant after induction (S), precipitate after induction (P), beads (B), flow through sample (FT), beads after wash (BAW), Elution sample (E), Beads after cutting lable (BAL). All the above samples were heated in 100°C metal bath for 3–5 min and centrifuged at 13000 rpm for 3–5 min for SDS-PAGE **(A)**. The protein BspF purified by SD200 after concentrating. Ordinate shows absorbance value of BspF at 260 nm (Red) and 280 nm (Blue) **(B)**.

Supplementary Figure S3 The motif enrichment heatmap of upstream and downstream amino acids of all identified modification sites. Red indicates that this amino acid is significantly enriched near the modification site, and green indicates that this amino acid is significantly reduced near the modification site.

Supplementary Figure S4 Mass accuracy distribution of mass spectrometry data. The score of peptide matched by spectrogram (indicating the reliability of the peptide identification) is inversely related to the distribution of mass error. Higher score means smaller error.

Supplementary Figure S5 Identified peptides length. Most identified peptides are distributed between seven and twenty amino acids, it conforms to the nomothetic approach based on trypsin digestion and higher energy collision induced dissociation (HCD). The distribution of identified peptides met the quality control requirements.
