# Supplementary figures and images for: Lysine Acylation Modification Landscape of Brucella abortus Proteome and its Virulent Proteins
Source: Front Cell Dev Biol. 2022 Mar 1;10:839822. doi: 10.3389/fcell.2022.839822 (PMC8921143; doi:10.3389/fcell.2022.839822)

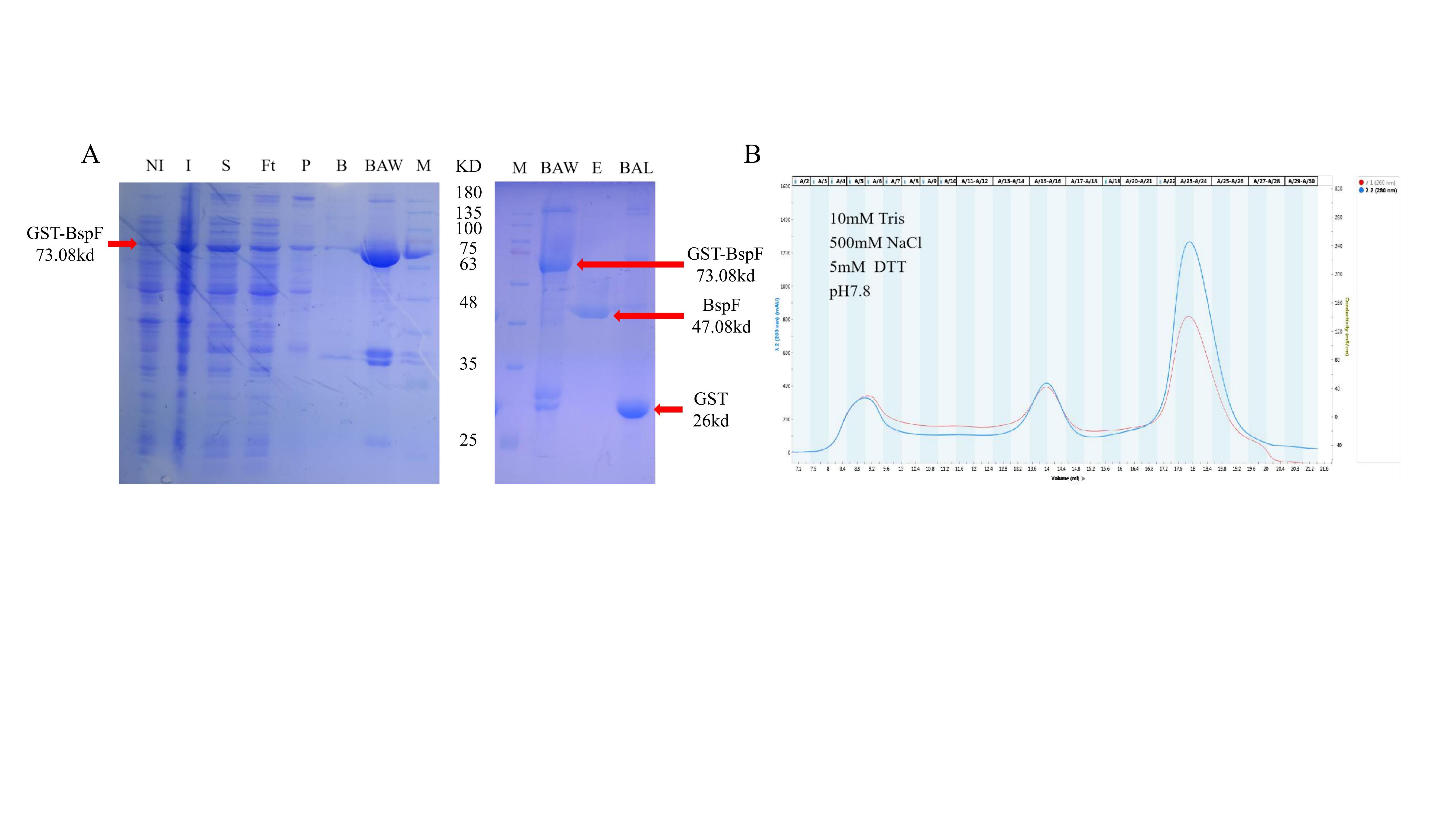

Supplement: Supplementary file 3 [file Image2.JPEG]

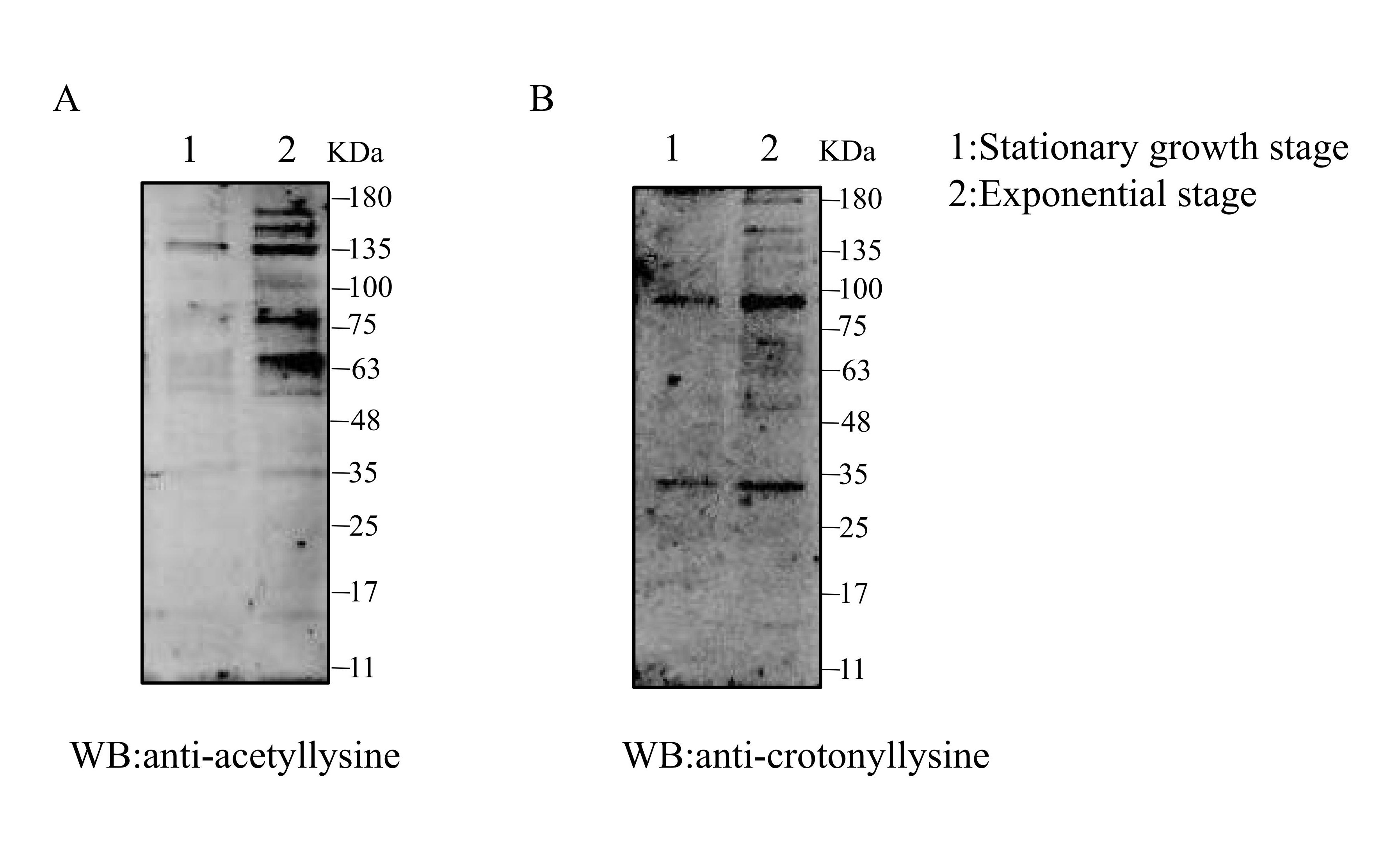

Supplement: Supplementary file 5 [file Image1.jpg]
